# Trend in the incidence of hepatitis A in mainland China from 2004 to 2017: a joinpoint regression analysis

**DOI:** 10.1186/s12879-022-07651-5

**Published:** 2022-08-01

**Authors:** Yuan-Sheng Li, Bei-Bei Zhang, Xi Zhang, Song Fan, Li-Ping Fei, Chao Yang, Ning-Jun Ren, Xiang Li, Ya-Mei Luo, Jun-Hui Zhang

**Affiliations:** 1 School of Public Health, Southwest Medical University, No.1, Section 1, Xianglin Road, Longmatan District, Luzhou, 646000 Sichuan People’s Republic of China; 2 Da’an District Center for Disease Control and Prevention, Zigong, 643010 Sichuan People’s Republic of China; 3 School of Medical Information and Engineering, Southwest Medical University, Luzhou, 646000 Sichuan People’s Republic of China; 4 Medicine & Engineering & Informatics Fusion and Transformation Key Laboratory of Luzhou City, Luzhou, 646000 Sichuan People’s Republic of China

**Keywords:** Hepatitis A, Joinpoint regression model, Incidence trend

## Abstract

**Background:**

China has experienced a continuous decreasing trend in the incidence of hepatitis A in recent years. Temporal trend analyses are helpful in exploring the reasons for the changing trend. Thus, this study aims to analyse the incidence trend of viral hepatitis A by region and age group in mainland China from 2004 to 2017 to evaluate the effectiveness of prevention and control measures.

**Methods:**

Data on hepatitis A and population information were collected and analysed with a joinpoint regression model. Annual percentage changes (APCs) and average annual percentage changes (AAPCs) were estimated for the whole country and for each region and age group.

**Results:**

From 2004 to 2017, the seasonality and periodicity of hepatitis A case numbers were obvious before 2008 but gradually diminished from 2008 to 2011 and disappeared from 2012–2017. The national incidence of hepatitis A (AAPC =  − 12.1%) and the incidence rates for regions and age groups showed decreasing trends, with differences in the joinpoints and segments. Regarding regions, the hepatitis A incidence in the western region was always the highest among all regions, while a nonsignificant rebound was observed in the northeastern region from 2011 to 2017 (APC = 14.2%). Regarding age groups, the hepatitis A incidence showed the fastest decrease among children (AAPC =  − 15.3%) and the slowest decrease among elderly individuals (AAPC =  − 6.6%). Among all segments, the hepatitis A incidence among children had the largest APC value in 2007–2017, at − 20.4%.

**Conclusion:**

The national annual incidence of hepatitis A continually declined from 2004 to 2017 and the gaps in hepatitis A incidence rates across different regions and age groups were greatly narrowed. Comprehensive hepatitis A prevention and control strategies, including the use of routine vaccination during childhood in mainland China, especially the implementation of the national Expanded Program on Immunization (EPI) in 2008, resulted in substantial progress from 2004 to 2017. However, gaps remain. Regular monitoring and analysis of hepatitis A epidemic data and prompt adjustment of hepatitis A prevention and control strategies focusing on children, elderly individuals and those living in certain regions are recommended.

## Background

Hepatitis A, caused by hepatitis A virus (HAV), is a self-limiting disease transmitted primarily via the faecal-oral route [1, 2] that does not usually cause chronic infection or chronic liver disease, and there is no specific treatment. Very few persons infected with hepatitis A die from fulminant hepatitis [3]; however, safe and effective vaccines are available to prevent hepatitis A [4]. While hepatitis A is widespread worldwide, the incidence of hepatitis A varies greatly among countries and regions with different socioeconomic statuses [5]. Over the past three decades, with the widespread administration of hepatitis A vaccines, economic and social development, improved sanitation, and guidance from a series of World Health Organization (WHO) documents on hepatitis A prevention and control, hepatitis A morbidity and mortality rates have decreased in the majority of countries and regions worldwide [6], and the average age of infection is increasing [7, 8]. As reported by the Global Burden of Disease 2017 (GBD 2017) study, the global numbers of hepatitis A cases and deaths in 2017 were approximately 170 million and 18.6 thousand, respectively [9, 10]. Currently, the regions with the highest disease burdens are developing countries in South Asia, sub-Saharan Africa, and Southeast Asia [11], indicating that hepatitis A is an important global public health problem [6].

Hepatitis A is a legal category B infectious disease and is a common cause of foodborne illness in China [12]. China has experienced several hepatitis A outbreaks and epidemics, resulting in a serious disease burden [5, 13], with high morbidity and mortality rates before 1992. To control the hepatitis A epidemic, China established a nationwide self-funded vaccination programme incorporating live attenuated hepatitis A vaccine and inactivated hepatitis A vaccine in 1992 and 2002 [14], respectively; this programme effectively reduced the population susceptible to hepatitis A but did not completely control the epidemic [7]. Therefore, in 2008, the hepatitis A vaccine was included in the national Expanded Program on Immunization (EPI) [15]. Vaccination together with the development of the economy and greater improvements in environmental sanitation effectively controlled the hepatitis A epidemic in China, and the epidemiological features of the hepatitis A incidence have changed dramatically [7]. From 1991–2017, the hepatitis A incidence showed a notable downward trend at the national level, with a decrease from 55.7/100,000 people in 1991 [16] to less than 2/100,000 people in 2017 [17], and the hepatitis A epidemic in China gradually shifted from a high to medium–low level [18], although it has not yet been completely interrupted.

It is beneficial to analyse hepatitis A surveillance data to assess the epidemiological characteristics and temporal trends of hepatitis A incidence in China to explore the common and specific causes of the change trends [19] and identify target populations and key measures for hepatitis A prevention and control. This will contribute to the formulation of more precise prevention and control strategies and the allocation of public health resources. Joinpoint regression analysis is a temporal trend analysis method that is superior to other temporal trend analysis methods that simply analyse overall trends, as it can divide a time period into several segments and find the statistically significant joinpoints of the temporal trend to reveal the features of local data [20]. Temporal trends in the incidence of hepatitis A surveillance data have not been fully evaluated in China, and there have been very few studies on the temporal trends of hepatitis A incidence using joinpoint regression models, especially by region and by demographic subgroup [18, 21]. Therefore, the purpose of this study was to analyse the incidence trends of hepatitis A by age group and region in China from 2004 to 2017 using joinpoint regression models.

## Methods

### Data source

We downloaded population-based surveillance data on the number of cases and incidence of hepatitis A in mainland China (abbreviated as China) from 2004 to 2017 for the total country and by province (municipalities or autonomous regions), age, month and year from the China Public Health Science Data Center, which covers 31 provinces in China (excluding Hong Kong, Macau and Taiwan). We compared the collected annual and monthly incidence data with the Centers for Disease Control (CDC) data and found them to be exactly the same. Demographic information was obtained from the China Statistical Yearbook.

### Study area and age groups

This study categorized the provinces/municipalities/autonomous regions of China into four regions based on the report "Strategies and Policies for Coordinated Regional Development" published in 2005 [22]: the less developed western region comprising Xinjiang, Ningxia, Yunnan, Guizhou, Guangxi, Chongqing, Gansu, Tibet, Sichuan and Qinghai; the central region comprising Henan, Shaanxi, Shanxi, Anhui, Hunan, Hubei, Jiangxi and Inner Mongolia; the highly developed eastern region comprising Hebei, Shandong, Tianjin, Beijing, Zhejiang, Jiangsu, Shanghai, Guangdong, Fujian and Hainan; and the northeastern region comprising Liaoning, Jilin and Heilongjiang Provinces.

In this study, we categorized the entire population into three age groups according to standard I-MOVE age groups [23], with those aged 0–14 years considered children, 15–64 years considered adults, and 65 and above considered elderly individuals.

### Statistical analysis

Excel 2019 was used to collect and organize the data, and SPSS Statistics 24.0 software (IBM, Armonk, NY, USA) was employed to test for normality and perform descriptive statistical analyses. Temporal trends in the national incidence of hepatitis A and the incidence by region and age group were measured by the annual percentage change (APC) and average annual percentage change (AAPC), with 95% confidence intervals (CIs), using joinpoint regression models [24]. We used a permutation procedure to determine the numbers of change-points (joinpoints) and estimate their locations and relevant regression parameters. When describing temporal trends, the terms increase and decrease indicate that the slope (APC and AAPC) was significant at the 0.05 level. The term stable denotes that the APC and AAPC were not significant (*p* ≥ 0.05) and indicates that the incidence remained at a stable level over the year. All P values were two-sided, with a significance level of 0.05. Joinpoint Regression Program 4.9.0.0. [25] was used to fit the joinpoint regression model, with a maximum of two joinpoints and three segments [26] according to the number of time points in this study. For the national temporal trend analysis, age-adjusted hepatitis A incidence rates were employed, using the age composition of our population in 2010 as the standard population, and crude hepatitis A incidence rates were analysed for temporal trends by region and age group with joinpoint regression models. In 2008, the hepatitis A vaccine was included in the national Expanded Programme on Immunization (EPI). Therefore, we analysed the data around this key timepoint.

## Results

### Results of the descriptive analysis

**Fig. 1:**
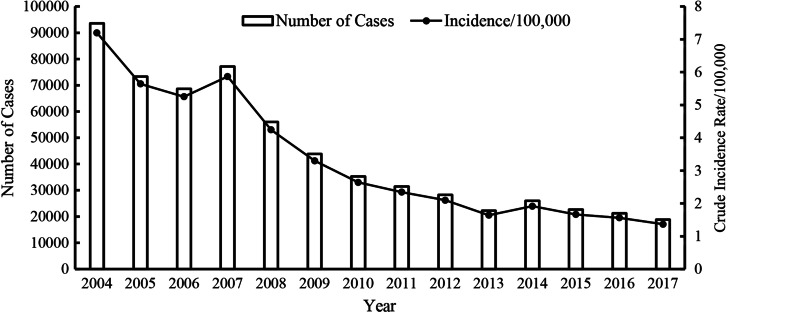
Yearly case numbers and crude incidence rates of hepatitis A in mainland China, 2004–2017

A total of 668,939 cases and 240 deaths due to hepatitis A were reported in China from 2004–2017, with an average annual incidence of 2.9055/100,000 people and a mortality rate of 0.0012/100,000 people. During the study period, there were overall decreasing trends in the national yearly case numbers and incidence rates of hepatitis A, with the incidence of hepatitis A decreasing from the highest value of 7.1996/100,000 people in 2004 to the lowest value of 1.3679/100,000 people in 2017, a decrease of 81.0%, as shown in Fig. 1.

### National seasonality, periodicity and temporal trend

**Fig. 2:**
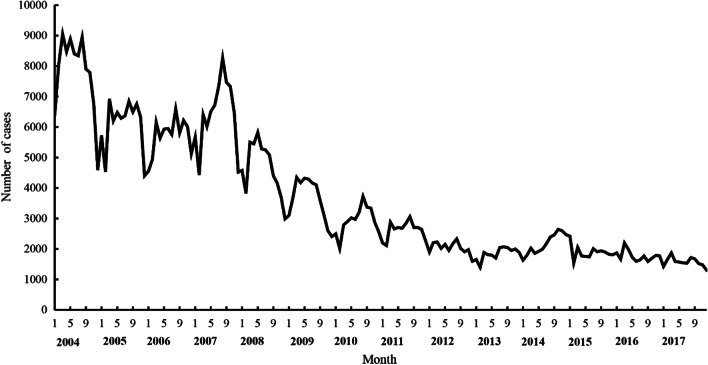
Monthly case numbers of hepatitis A in mainland China, 2004–2017

As shown in Fig. 2, from 2004 to 2007, the hepatitis A case numbers showed distinct seasonality and periodicity, with a higher number of cases in 2004 and 2007 than in other years *and seasonal peaks* from March to August of each year, with the lowest case numbers in February. The seasonality and periodicity of hepatitis A case numbers gradually diminished from 2008 to 2011 and were no longer obvious from 2012 to 2017; there were relatively stable and low numbers of monthly hepatitis A cases from 2012 to 2017.

**Fig. 3:**
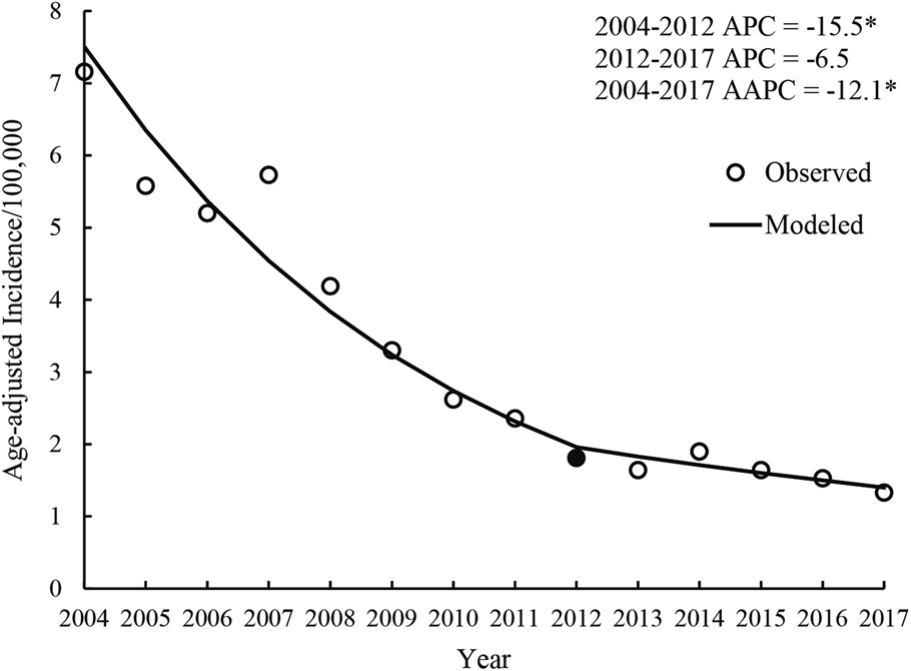
Yearly age-adjusted incidence rates of hepatitis A in mainland China during 2004–2017 based on the joinpoint regression model. *Indicates *p* < 0.05. The solid dot indicates a joinpoint (turning point demarking significance). The age composition of the Chinese population in 2010 was used as the standard population. *AAPC* average annual percentage change, *APC* annual percentage change

The result of the joinpoint regression analysis of the temporal trend of hepatitis A is presented in Fig. 3. The national age-adjusted incidence of hepatitis A in China showed a decrease from 2004 to 2017 (AAPC = − 12.1%; 95% CI, − 15.0–− 9.2; *p* < 0.001), with a minor rebound in 2007 and 2014. Additionally, the trend had a joinpoint corresponding to the year 2012, with a rapid decrease from 2004 to 2012 (APC = − 15.5%; 95% CI, − 18.6–− 12.2; *p* < 0.001) and a stable, moderate decrease from 2012 to 2017 (APC = − 6.5%; 95% CI, − 13.5–1.0; *p* = 0.081).

### Temporal trends by regions

**Fig. 4:**
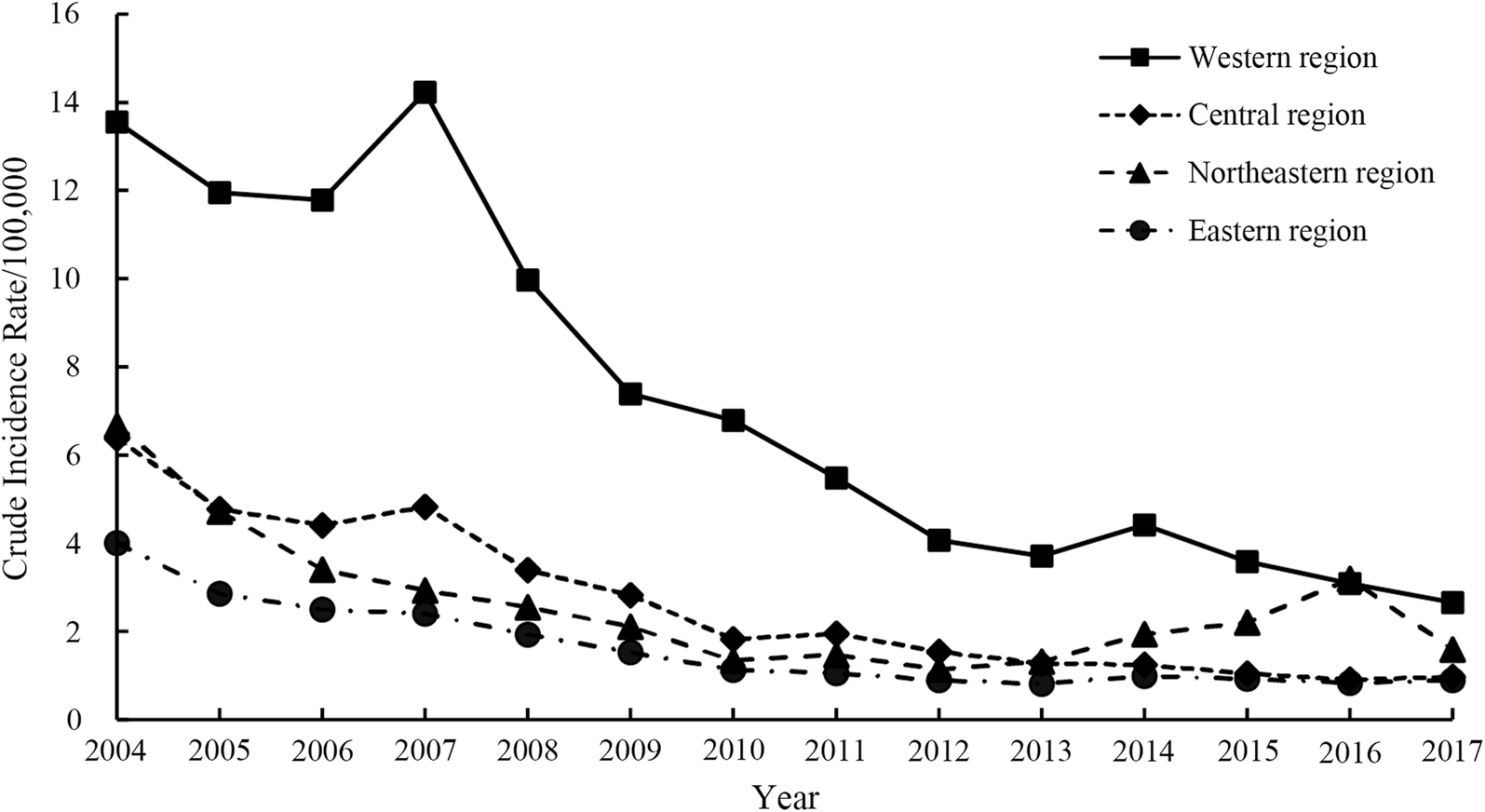
Yearly crude incidence rates of hepatitis A in mainland China by region, 2004–2017

As illustrated in Fig. 4, the incidence of hepatitis A in China displayed decreasing trends in all four regions throughout the study period. The western region had the highest incidence and a greater degree of decline, whereas the central and eastern regions had steady yearly downward trends. In the northeastern region, the incidence rebounded from 1.14/100,000 people in 2012 to 3.19/100,000 people in 2016 but decreased to less than 2/100,000 people in 2017.

**Fig. 5:**
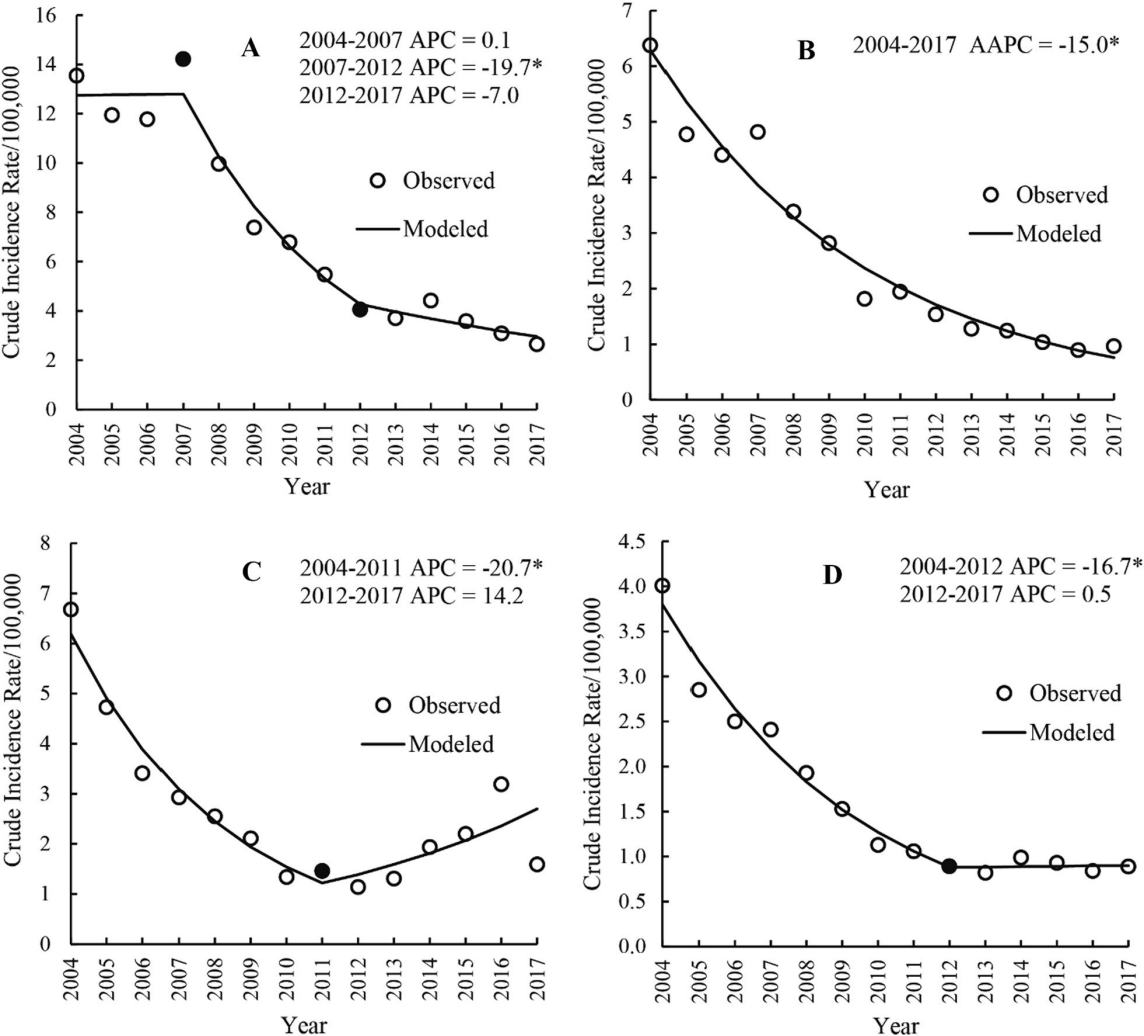
Trends in the yearly incidence rates of hepatitis A in four regions in mainland China during 2004–2017 based on the joinpoint regression model. *Indicates *p* < 0.05. Solid dots indicate joinpoints (turning point demarking significance). *AAPC* average annual percentage change, *APC* annual percentage change.** A**,** B**,** C** and** D** represent the western, central, northeastern and eastern regions, respectively

The results of the joinpoint regression analysis (Table 1) revealed that the incidence of hepatitis A in the western region showed an overall decrease (AAPC =  − 10.6%; 95% CI, − 14.3– − 6.8; *p* < 0.001), with a stable trend in 2004–2007 (APC = 0.1%; 95% CI, − 9.5–10.7; *p* = 0.984), a rapid decline in 2007–2012 (APC =  − 19.7%; 95% CI, -26.2– − 12.7; *p* = 0.001), and a gradual decline in 2012–2017 (APC =  − 7.0%; 95% CI, − 14.9–1.6; *p* = 0.090) (Fig. 5A).

**Table 1:** Relative changes in the yearly incidence rates of hepatitis A in different regions in mainland China from 2004 to 2017 based on joinpoint regression analysis

Regions	Duration	APC and 95% CI (%)	*p*-value	AAPC and 95% CI (%)	*p*-value
Western region	2004–2007	0.1 (− 9.5 to 10.7)	0.984	− 10.6 (− 14.3– − 6.8)*	< 0.001
	2007–2012	− 19.7 (− 26.2– − 12.7)*	0.001		
	2012–2017	− 7.0 (− 14.9–1.6)	0.090		
Central region	2004–2017	− 15.0 (− 16.8– − 13.2)*	< 0.001	− 15.0(− 16.8– − 13.2)*	< 0.001
Northeastern region	2004–2011	− 20.7 (− 27.0– − 14.0)*	< 0.001	− 6.2(− 12.5–0.5)	0.070
	2011–2017	14.2 (− 1.2–31.9)	0.068		
Eastern region	2004–2012	− 16.7 (− 19.0– − 14.4)*	< 0.001	− 10.5(− 13.2– − 7.7)*	< 0.001
	2012–2017	0.5 (− 7.4–9.1)	0.891		

*Indicates *p* < 0.05

In the central region, there was an overall decrease in the hepatitis A incidence throughout the study period (AAPC =  − 15.0%; 95% CI, − 16.8– − 13.2; *p* < 0.001), with no joinpoint from 2004–2017 (Fig. 5B).

In the northeastern region, the overall hepatitis A incidence trend showed a nonsignificant gradual decline throughout the study period (AAPC =  − 6.2%; 95% CI, − 12.5–0.5; *p* = 0.070), with a significant rapid decline from 2004 − 2011 (APC =  − 20.7%; 95% CI, − 27.0– − 14.0; *p* < 0.001) and a nonsignificant gradual increase from 2011–2017 (APC = 14.2%; 95% CI, − 1.2 − 31.9; *p* = 0.068) (Fig. 5C).

In the eastern region, there was an overall decrease in the incidence of hepatitis A (AAPC =  − 10.5%; 95% CI, − 13.2– − 7.7; *p* < 0.001), with a significant decline from 2004 to 2012 (APC =  − 16.7%; 95% CI, − 19.0– − 14.4; *p* < 0.001) and a stable trend from 2012 to 2017 (APC = 0.5%; 95% CI, − 7.4–9.1; *p* = 0.891) (Fig. 5D).

### Temporal trend by age group

**Fig. 6:**
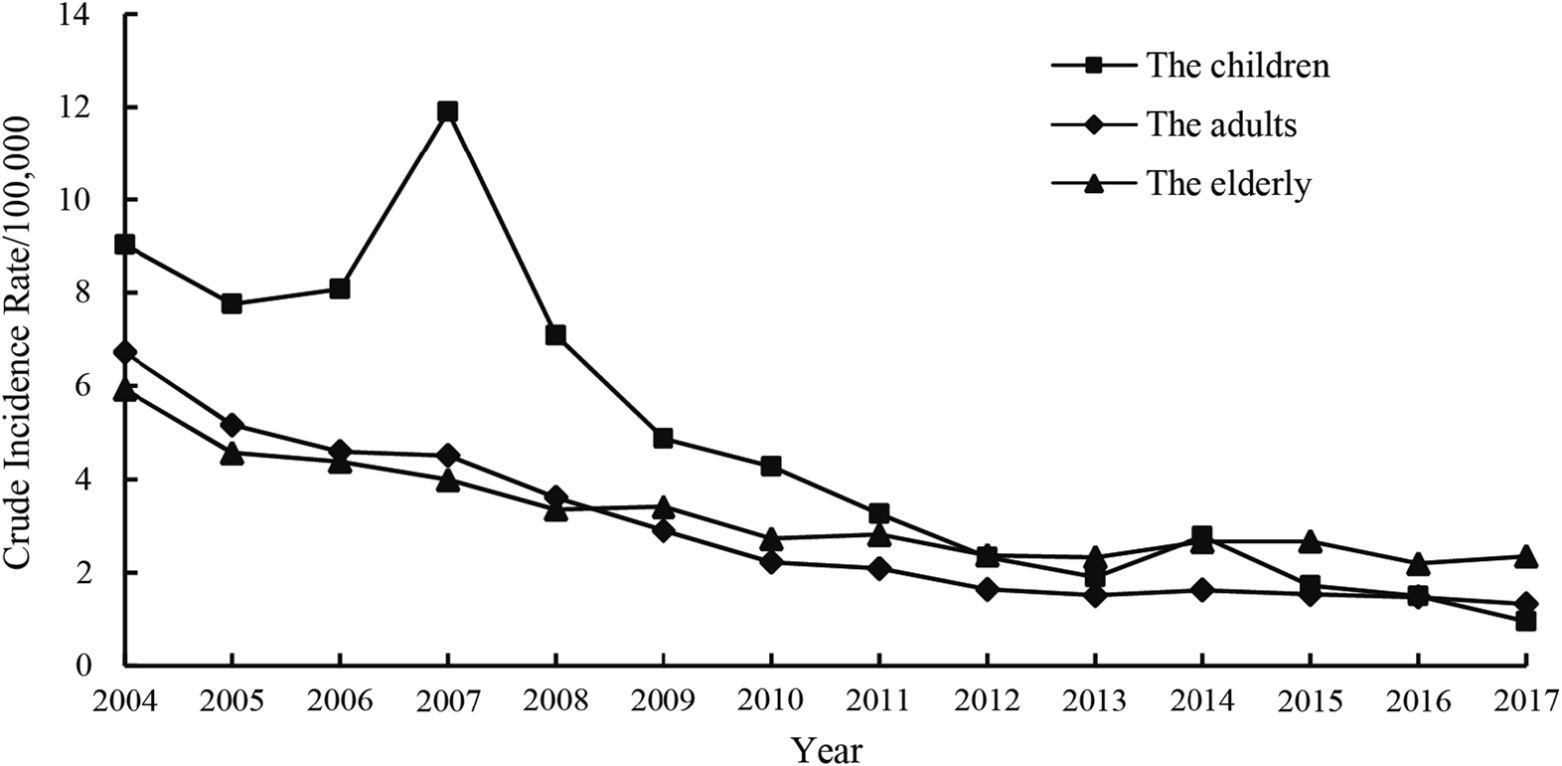
Yearly crude incidence rates of hepatitis A in all age groups in mainland China, 2004–2017

Overall, from 2004 to 2017, the incidence of hepatitis A in China declined in all age groups, with the fastest decline among children from the highest incidence in 2004, at 9.03/100,000, to the lowest incidence in 2017, at 0.95/100,000. The elderly population had the slowest decline, from the highest incidence of 5.94/100,000 in 2004 to the lowest incidence of 2.35/100,000 in 2017. The incidence among adults showed a steady decline (Fig. 6).

**Fig. 7:**
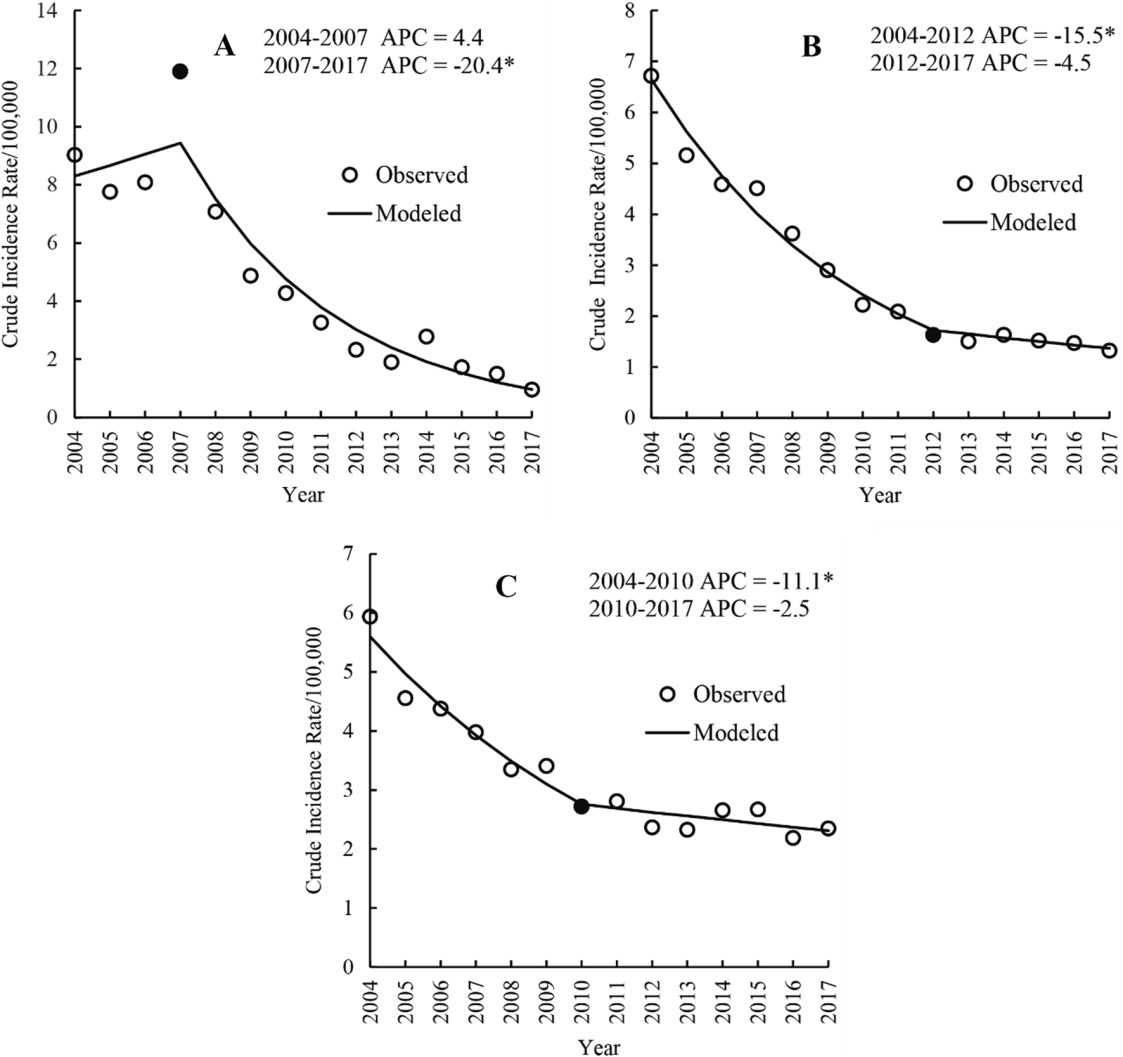
Trends in the yearly incidence rates of hepatitis A before/after the EPI in age groups in mainland China during 2004–2017 based on the joinpoint regression model. Note: In 2008, hepatitis A vaccine was included in the national Expanded Programme on Immunization. *Indicates *p* < 0.05. Solid dots indicate joinpoints (turning point demarking significance). *APC* annual percentage change.** A**,** B**, and** C** represent the 0–14 group, 15–64 group and ≥ 65 group, respectively

The joinpoint regression analyses revealed significant incidence decreases among children (AAPC = − 15.3%; 95% CI, − 18.7–− 11.7; *p* < 0.001), adults (AAPC = − 11.5%; 95% CI, − 13.9–− 9.0; *p* < 0.001) and elderly individuals (AAPC = -6.6%; 95% CI, − 8.7–− 4.4; *p* < 0.001) throughout the study period. One significant joinpoint was detected in each age group (Table 2 and Fig. 7A–C). Among children, the incidence of hepatitis A remained stable from 2004 to 2007 (APC = 4.4%; 95% CI, − 9.92–21.0; *p* = 0.525) and showed a significant rapid decrease from 2007 to 2017 (APC =  − 20.4%; 95% CI, − 23.8– − 16.8; *p* < 0.001). Among adults, the incidence decreased rapidly from 2004 to 2012 (APC =  − 15.5%; 95% CI, − 17.5– − 13.5; *p* < 0.001) and remained stable from 2012 2017 (APC =  − 4.5%; 95% CI, − 11.4–2.9; *p* = 0.194). In the elderly population, the incidence exhibited a significant downward trend in 2004–2010 (APC =  − 11.1%; 95% CI, − 14.6– − 7.5; *p* < 0.001) and remained stable in 2010–2017 (APC =  − 2.5%; 95% CI, − 6.0 − 1.1; *p* = 0.152).

**Table 2:** Relative changes in the yearly incidence rates of hepatitis A before/after the EPI in different age groups in mainland China from 2004 to 2017 based on joinpoint regression analysis

Age group(year)	Duration	APC and 95%CI (%)	*p*-value	AAPC and 95%CI (%)	*p*-value
0–14	2004–2007	4.4 (− 9.9–21.0)	0.525	− 15.3 (− 18.7– − 11.7)*	< 0.001
	2007–2017	− 20.4 (− 23.8– − 16.8)*	< 0.001		
15–64	2004–2012	− 15.5 (− 17.5– − 13.5)*	< 0.001	− 11.5 (− 13.9– − 9.0)*	< 0.001
	2012–2017	− 4.5 (− 11.4–2.9)	0.194		
≥ 65	2004–2010	− 11.1 (− 14.6– − 7.5)*	< 0.001	− 6.6 (− 8.7– − 4.4)*	< 0.001
	2010–2017	− 2.5 (− 6.0–1.1)	0.152		

In 2008, the hepatitis A vaccine was included in the national Expanded Programme on Immunization. *Indicates p < 0.05

## Discussion

We reported the national temporal trend of the hepatitis A incidence, and incidence trends by region and age group. We also analysed incidence seasonality and periodicity based on hepatitis A surveillance data from 2004 to 2017 in China. In this study, a significant downward trend in the national incidence of hepatitis A as well as in the annual incidence of hepatitis A in all age groups and in all regions during the study period was observed, indicating that prevention and control measures across the country have been successful in recent years; this was consistent with the results of other descriptive studies of hepatitis A incidence [7, 27]. Throughout the study period, the hepatitis A incidence varied among different regions, with the highest incidence in the western region compared to the other three regions. Similarly, previous studies found that the incidence rates among children under the age of 10 in the provinces of Xinjiang, Qinghai, and Tibet were high [7, 16]. Notably, the incidence of hepatitis A began to increase in the northeastern region in 2012 and increased to a relatively high level in 2016. This result was similar to that in other studies in which the only region with a rebound in hepatitis A incidence in recent years was Liaoning. This increase was in accordance with that of the northeastern region, probably due to the ingestion of contaminated raw or semiraw seafood by residents in some coastal cities in the northeastern region [28, 29].

The seasonality and periodicity patterns of the national hepatitis A case numbers observed in this study are supported by previous studies [7, 30]. These patterns were probably due to the addition of the hepatitis A vaccine to the EPI in 2008 [15]. The widespread administration of the vaccine has changed the epidemiological characteristics of hepatitis A and eliminated the seasonal peaks of hepatitis A incidence [31, 32] However, some studies have shown that hepatitis A still occurs periodically in some provinces, including Xinjiang in the western region [33] and Liaoning in the northeastern region [34], and seasonal peaks are more likely due to the consumption of contaminated food or water or infrequent hand washing [35, 36]. Therefore, it is recommended that when the national hepatitis A epidemic is at a low level, an analysis of the seasonal characteristics of the epidemic in certain regions be carried out to guide regional targeted prevention and control measures before the epidemic season.

The results of the joinpoint regression model showed that there were differences in the joinpoints and downward trends of segments in the temporal trends of the hepatitis A incidence for the country overall and in each region. From a national perspective, the joinpoint regression model detected one joinpoint (2012) in the hepatitis A incidence, with a rapid decline from 2004 to 2012 and a plateau from 2012 to 2017. In terms of regions, the joinpoint regression model detected different joinpoints and segments for each region; this was mainly because different regions in China implemented the EPI at different times and with different intensities, suggesting that there might be gaps in EPI-based vaccination against hepatitis A. Accordingly, more in-depth research and precise interventions are needed. For example, in 2014, hepatitis A vaccine coverage among children aged 2–14 years in Shanghai was high, at 99.9%, while in some western regions, such as Tibet, where health care resources and public health awareness are insufficient [37], it was lower, at only 76.1% [38]. Another reason might be that the different economic levels of each region were negatively correlated with the incidence of hepatitis A [7]. Therefore, the hepatitis A incidence in the western region, which has a lower economic level, was higher before the implementation of the EPI. The socioeconomic inequality associated with hepatitis A vaccine coverage was nearly eliminated with the implementation of the EPI [7, 39]; thus, the decreasing hepatitis A incidence trend in the western region was more significant than in other regions with higher economic levels. Other possible reasons for regional disparities in hepatitis A incidence rates include discrepancies in sanitary conditions [40], hygiene habits and health awareness among populations in different regions [18]. Therefore, conducting special studies in high hepatitis A incidence areas, such as Liaoning and Tibet, to determine the underlying causes and establish targeted measures to reduce the risk of hepatitis A infection is advised.

Regarding age groups, the results of the joinpoint regression model showed a decreasing trend in the incidence of hepatitis A in all age groups in China from 2004 to 2017. For children, the joinpoint coincided with the time point of the issuance of the EPI in 2008 in China; this indicated that the fastest decreasing trend in the hepatitis A incidence from 2008–2017 was mainly attributed to the implementation of the EPI in 2008, which targeted all children over the age of 18 months [15], and resulted in a shift in the hepatitis A incidence among children from the highest incidence in 2004 to the lowest incidence in 2017 [16, 26]. However, other studies have shown that the hepatitis A incidence in children aged 0–4 years was still relatively high in 2017 [11, 20], possibly due to low hepatitis A vaccination coverage among children in some regions, the reasons for which might include an inadequate supply of vaccines [41, 42]. Thus, there is a need to further investigate hepatitis A vaccination coverage and hepatitis A antibody levels among children in each region in the future to more precisely identify weak points in prevention and control measures. Among elderly individuals, the incidence showed a relatively slow downward trend compared to the other two age groups, from the lowest level in 2004 to the highest level in 2017. This might be due to an indirect protective effect of the EPI [7]; even though elderly individuals were not the target population for the vaccine, the vaccination of those around them potentially resulted in a relatively decreased rate of seropositivity in this group, which is supported by evidence from serological antibody studies [43]. In addition, other potential reasons for the slow decline and relatively high incidence of hepatitis A in the elderly population include weakened immunity [44], an increase in the susceptible population [32], and community transmission [45, 46]. Additional reasons for the decline in the hepatitis A incidence in all age groups include increased economic levels and better sanitation [5, 7, 17], which are supported by other studies [7, 38, 39].

As mentioned above, comprehensive prevention and control measures including the use of routine vaccination during childhood in mainland China, especially the EPI in 2008, resulted in a rapid downward trend in the hepatitis A incidence rate nationwide before 2012, and the gaps in hepatitis A incidence rates across different regions and age groups were narrowed. Notably, after 2012, the hepatitis A incidence rates nationwide, by region and by age group decreased to a plateau and even increased in some regions, probably because the incidence rates of hepatitis A in different regions and age groups were already at relatively low levels, with limited potential for further decline. The specific reasons for the plateau need to be investigated further. In addition, the gaps in hepatitis A incidence rates among different regions and age groups remain, especially the western region and the elderly group, which have relatively high incidence rates. These gaps suggest that the prevention and control of hepatitis A should focus on the western region and the elderly group to further reduce the national hepatitis A incidence level.

Therefore, our suggestions for the future prevention and control of hepatitis A in China comprise comprehensive prevention and control measures, focusing on hepatitis A vaccination, in accordance with the Action Plan for the Prevention and Treatment of Viral Hepatitis in China (2017–2020); these measures consolidate the current achievements of hepatitis A prevention and control and further reduce the hepatitis A incidence [47]. The specific strategies and measures are as follows: the expansion of hepatitis A vaccination coverage to more groups, the promotion of economic development in each region, and the timely supply of hepatitis A vaccines and the strict implementation of vaccination among children. Moreover, people who did not initially receive hepatitis A vaccination should be immediately vaccinated to narrow the gaps between the immunization levels in different age groups [43]. Since some studies have shown that the seropositivity rate of hepatitis A among elderly individuals is 65% [2], it is recommended that persons not covered by the EPI, especially elderly persons, undergo voluntary vaccination. Other measures include enhancement of public health education and improvement of sanitary conditions, especially ensuring access to high-quality drinking water in rural areas. Finally, it is advised to regularly monitor and analyse the epidemiological characteristics of hepatitis A in different populations, especially those in areas and age groups with a high incidence of hepatitis A, and promptly adjust prevention and control strategies according to epidemic dynamics, with a particular emphasis on children, elderly individuals and persons living in certain regions.

There are several limitations to our study. First, due to limited data access privileges, we could obtain data only on hepatitis A incidence by age group and by region; we could not access additional demographic data in each region during 2004–2017, so we could not analyse the temporal trends of hepatitis A incidence in subgroups according to other demographic factors in specific years and regions. Second, at the time of data collection in this study, only hepatitis A data from 2004 to 2017 were available for download. Third, the Public Health Science Data Center is a passive surveillance system, and data on hepatitis A were influenced by factors such as residents' consultation behaviours, the diagnostic levels of medical institutions, and the completeness and accuracy of the reported data; therefore, the incidence in certain regions might be underestimated. It is necessary to further improve the quality of disease surveillance in all regions. Finally, temporal trend analysis cannot explain the direct causes of changes in hepatitis A incidence, and it is necessary to conduct further research on the causes of these different trends.

## Conclusion

In conclusion, the national annual incidence of hepatitis A in China continually declined from 2004 to 2017, and the gaps in hepatitis A incidence rates across different regions and age groups were greatly narrowed. The decreasing temporal trends of the hepatitis A incidence coincided with the implementation of the EPI in China in 2008, especially among children; this indicated that China's comprehensive hepatitis A prevention and control strategies based on vaccines achieved great success. However, gaps remain. It is recommended to regularly monitor and analyse hepatitis A vaccination coverage and epidemic levels in various age groups in each region; promptly adjust the hepatitis A vaccination strategy; and adopt individualized and precise prevention and control policies for specific populations, focusing on children, elderly individuals, and those living in certain regions. The key measures for hepatitis A prevention and control are to promote vaccination coverage, improve sanitary conditions, and provide high-quality drinking water in rural areas.

## Data Availability

The datasets supporting the conclusions of this article are publicly available from the Data-Center of China Public Health Science of National Population and Health Science Data Sharing Platform (https://www.phsciencedata.cn/Share/en/index.jsp).
